# Predicting Relapsing-Remitting Dynamics in Multiple Sclerosis Using Discrete Distribution Models: A Population Approach

**DOI:** 10.1371/journal.pone.0073361

**Published:** 2013-09-05

**Authors:** Nieves Velez de Mendizabal, Matthew M. Hutmacher, Iñaki F. Troconiz, Joaquín Goñi, Pablo Villoslada, Francesca Bagnato, Robert R. Bies

**Affiliations:** 1 Indiana University School of Medicine; Indianapolis, Indiana, United States of America; 2 Indiana Clinical and Translational Sciences Institute (CTSI), Indianapolis, Indiana, United States of America; 3 Ann Arbor Pharmacometrics Group (A2PG), Ann Arbor, Michigan, United States of America; 4 Department of Pharmacy and Pharmaceutical Technology, School of Pharmacy, University of Navarra, Pamplona, Spain; 5 Department of Psychological and Brain Sciences, Indiana University, Bloomington, Indiana, United States of America; 6 Center for Neuroimmunology, Institute of Biomedical Research August Pi Sunyer (IDIBAPS), Hospital Clinic of Barcelona, Barcelona, Spain; 7 Neuroimmunology Branch, National Institute of Neurological Disorders and Stroke, NIH, Bethesda, Maryland, United States of America; 8 Department of Neurology, University of Maryland, Baltimore, Maryland, United States of America; University of Jaén, Spain

## Abstract

**Background:**

Relapsing-remitting dynamics are a hallmark of autoimmune diseases such as Multiple Sclerosis (MS). A clinical relapse in MS reflects an acute focal inflammatory event in the central nervous system that affects signal conduction by damaging myelinated axons. Those events are evident in T1-weighted post-contrast magnetic resonance imaging (MRI) as contrast enhancing lesions (CEL). CEL dynamics are considered unpredictable and are characterized by high intra- and inter-patient variability. Here, a population approach (nonlinear mixed-effects models) was applied to analyse of CEL progression, aiming to propose a model that adequately captures CEL dynamics.

**Methods and Findings:**

We explored several discrete distribution models to CEL counts observed in nine MS patients undergoing a monthly MRI for 48 months. All patients were enrolled in the study free of immunosuppressive drugs, except for intravenous methylprednisolone or oral prednisone taper for a clinical relapse. Analyses were performed with the nonlinear mixed-effect modelling software NONMEM 7.2. Although several models were able to adequately characterize the observed CEL dynamics, the negative binomial distribution model had the best predictive ability. Significant improvements in fitting were observed when the CEL counts from previous months were incorporated to predict the current month’s CEL count. The predictive capacity of the model was validated using a second cohort of fourteen patients who underwent monthly MRIs during 6-months. This analysis also identified and quantified the effect of steroids for the relapse treatment.

**Conclusions:**

The model was able to characterize the observed relapsing-remitting CEL dynamic and to quantify the inter-patient variability. Moreover, the nature of the effect of steroid treatment suggested that this therapy helps resolve older CELs yet does not affect newly appearing active lesions in that month. This model could be used for design of future longitudinal studies and clinical trials, as well as for the evaluation of new therapies.

## Introduction

Multiple sclerosis (MS) is a prototypic autoimmune disease that affects the central (CNS) with a relapsing-remitting (RR) disease progression [1]. Clinical relapses in MS, acute symptoms that appear in episodic periods, are considered to be the reflection of focal inflammatory events in the white matter that disrupts neural conduction by damaging axons [2]. Clinical relapses are used to categorize different forms of the disease, i.e. RR versus progressive MS, as a marker to define the disease's disease progression and to measure the success of new therapies [2].

**Figure 1:**
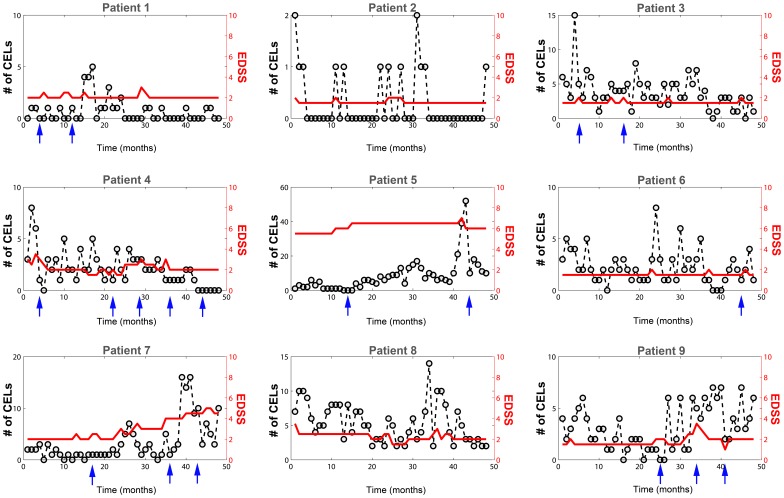
Number of contrast-enhancing lesions (CELs). CEL counts are represented with circles and dashed lines (left Y axis). Some patients were treated with intravenous methylprednisolone at 1 g/day for 3–5 days, or oral prednisone taper for clinical relapses (arrows). Changes in the EDSS are plotted on the right Y axis (red line).

Magnetic Resonance Image (MRI) is a useful tool for understanding and following the disease progression in patients with MS [3]–[5]. The focal inflammatory events of the CNS that accompany a clinical MS relapse are evident on MRI recordings as contrast enhancing lesions (CELs) on T1-weighted images [6]. This kind of MRIs shows CELs four to ten times more frequently compared with clinically defined relapses [7]. That is, clinical relapses may not occur even if a CEL is observed. Therefore, CELs are more informative biomarker for disease progression than the Expanded Disability Status Score (EDSS). The natural history of a CEL is highly variable both within and between patients (Figure 1). In MS, CELs and associated clinical relapses generally last for a month with spontaneous partial or full recovery afterwards. The CEL distribution over time has not been associated with any specific pattern or cause to date [2], [8]. However, in one third of cases, relapses are preceded by either a stressful events and/or infections [9], [10].

The number of CELs measured every month is a discrete response variable that can take only non- negative integer values (Figure 1). Modelling such count data has been applied to different processes including anticonvulsant responses [11], [12], incontinence [13], neonatal apnea [14] and epileptic seizures [15], [16]. Commonly the Poisson distribution (PS) model is used to describe the data. The mean counts in an arbitrary time interval for the PS model can be denoted as λ which can be influenced by several factors as drug effect, covariates (sex, weight, age…), disease progression, etc. The PS model has two restrictions: the mean (λ) is equal to the variance of the data and the numbers of events occurring in non-overlapping intervals of time are assumed independent. This is a significant challenge as many counting outcomes show *(i)* bigger or smaller variability than that predicted by the Poisson model, a phenomenon called over-dispersion or under-dispersion respectively and *(ii)* lack of independence in the counts observed in previous intervals. Therefore, discrete distribution models other that the Poisson should be explored to evaluate this heterogeneity along with the evaluation of Markovian elements to adjust for correlation in counts between intervals. Identification of models that better characterize the distribution of CELs is relevant for two reasons. First, it provides a predictive framework for the relapsing-remitting dynamic observed in patients with MS. Second, it can serve to inform the design of longitudinal studies of this disease. While several count distributions have been proposed to model this type of data, the negative binomial (NB) distribution has been consistently found to provide one of the better fits to the data [17]–[20]. Although the NB has already demonstrated a very good fitting with this kind of count data, it might be the case that the election of a smaller interval period for the MRI acquisitions had produced a different analysis outcome. The best scanning interval and analysis for this outcome is not clear since monthly scans generally provided more prediction power but they are more expensive [21]. In this study we analysed the distribution of CELs developed by nine MS patients whom underwent monthly MRI for 48 months. Here we used the unique high resolution of this dataset for the exploration of other distributions adding other factors that might effect changes in the disease dynamic. Concretely, Markovian effects on the model parameters have been explored and quantified. In addition, how corticosteroids affect the lesions counts was also included during the model exploration and development. The short interval between MRI acquisitions (one month) shows an adequate time resolution to capture the relapsing-remitting dynamics of this disease.

## Results

**Figure 2:**
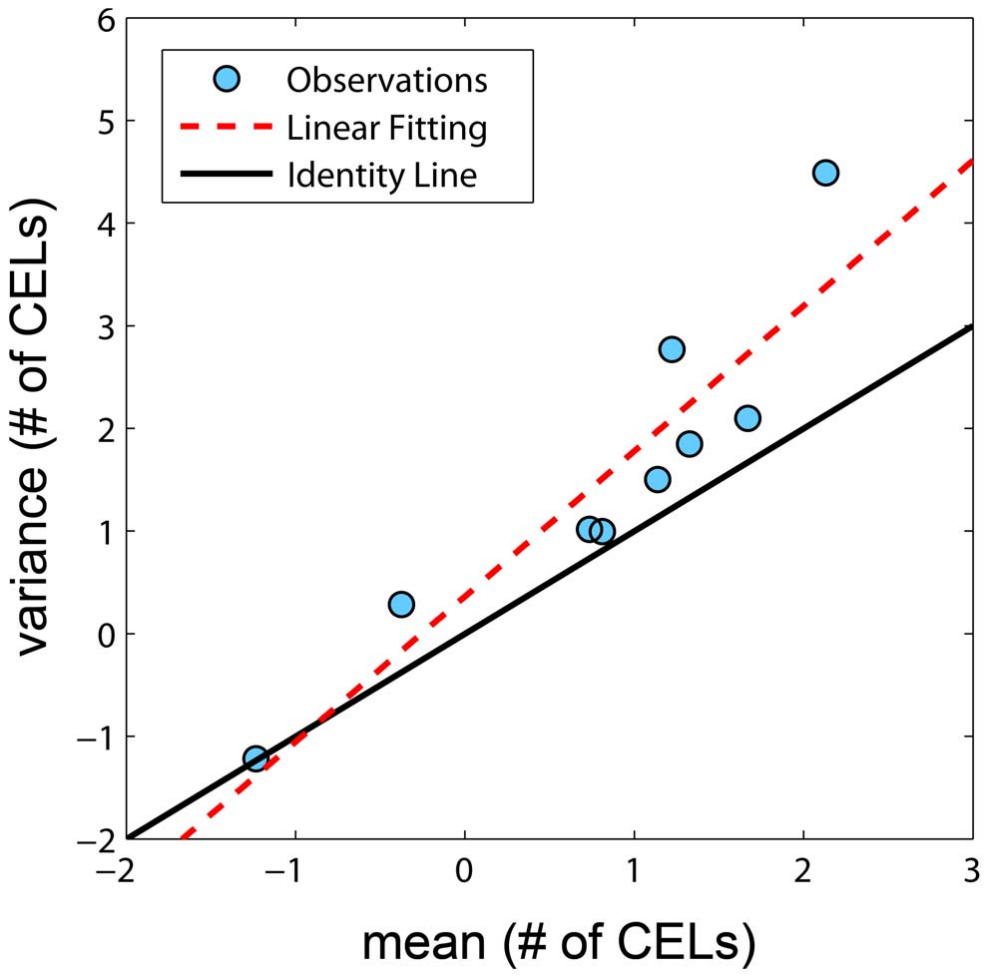
Variance *versus* mean of number of CELs obtained from the raw data. Each observation represents one patient and is represented by dots. Solid black line represents the identity line. Dashed red line is the linear data fit.

Several models were evaluated, resulting in sixteen key structural models based on seven different probability distributions. Based on the number of model parameters, the objective function (table 1) and the precision of the parameters the best fitting distribution was the negative binomial (equation 10) overcoming the other explored distributions like (Poisson model, Poisson model with mixture distribution, Zero-Inflated and Generalized Poisson models and the Zero-Inflated Negative Binomial model). When the variance and the mean of number of CELs were calculated from the raw data by subject, the variances were greater in magnitude than the means for all but 1 patient (Figure 2). A statistically significant difference in the objective function value (OFV) was observed when a first order Markov parameter θ_PDV_, which modified the mean counts (λ) parameter based on the counts observed from the previous MRI, was incorporated (*NB MAK2 model*, equation 4). When a second order Markov parameter θ_PPDV_ was included (*NB nested MAK2 model,* equation 5) relating the current mean count to the observed count two months prior, the statistical difference also significant, but the magnitude of the second order effect was less (θ_PDV_>θ_PPDV_ ). The same decreasing pattern, θ_PDV_>θ_PPDV_>θ_PPPDV_, was observed although the fit improvement was not significant when a third order Markov parameter was also included (*NB nested nested MAK2 model,* equation 6). Therefore, the best model was the negative binomial model (equation 10) with first and second order Markov parameters (equation 5): *NB nested MAK2 model*.

**Table 1:** Summary of the discrete-distribution models evaluated.

MODELS	PARAMETERS								−2LL(Δ model)
**PS**	**θ_λ_**	**ω_λ_**							2068.725
	0.744	0.442							
**PMAK1**	**θ_λ1_**	**θ_λ2_**	**ω_λ1_**						2001.0 (−67.55 **PS**)
	0.932	2.76	0.542						
**PMAK2**	**θ_λ0_**	**θ_PDV_**	**ω_λ0_**	**ω_PDV_**					1725.51 (−343.212 **PS**)
	1.18	0.418	0.562	0.187					
**nested PMAK2**	**θ_λ0_**	**θ_PDV_**	**θ_PPDV_**	**ω_λ0_**	**ω_PPDV_**				1713.88 (−11.63 **PMAK2**)
	1.03	0.388	0.124	0.501	0.164				
**nested nested PMAK2**	**θ_λ0_**	**θ_PDV_**	**θ_PPDV_**	**θ_PPPDV_**	**ω_λ0_**	**ω_PDV_**			1711.49 (−14.02 **PMAK2**)
	0.956	0.396	0.0974	0.0595	0.487	0.143			
**PMIX**	**θ_λ1_**	**θ_λ2_**	**θ_PM_**	**ω_λ1_**	**ω_λ2_**				1867.23 (+141.72 **PMAK2** )
	2.72	1.81	0.413	0.529	1.89				
**ZIP**	**θ_λ1_**	**θ_P0_**	**ω_λ1_**						2036.40 (+310.49 **PMAK2**)
	2.4	0.0375	0.91						
**GP**	**θ_λ_**	**θ_disp_**	**ω_λ_**						1808.41 (+82.9 **PMAK2**)
	1.53	0.393	0.663						
**GP_MAK2**	**θ_λ0_**	**θ_disp_**	**θ_PDV_**	**ω_λ0_**	**ω_PDV_**				1665.50 (−60.01 **PMAK2**)
	0.902	0.371	0.232	0.451	0.0932				
**GP_nested_MAK2**	**θ_λ0_**	**θ_disp_**	**θ_PDV_**	**θ_PPDV_**	**ω_λ0_**	**ω_PDV_**			1654.96 (−70.55 **PMAK2**)
	0.742	0.347	0.121	0.23	0.365	0.058			
NB	θ_λ_	θ_OVDP_	ω_λ_	ω_OVDP_					1758.92 (+33.41 PMAK2)
	2.32	0.254	0.898	0.829					
**ZINB**	**θ_λ_**	**θ_OVDP_**	**θ_P0_**	**ω_λ_**	**ω_OVDP_**				1758.63 (+33.12 **PMAK2**)
	2.32	0.254	0	0.898	0.829				
**NB_MAK2**	**θ_λ_**	**θ_OVDP_**	**θ_PDV_**	**ω_λ_**	**ω_PDV_**				1642.85 (−82.66 **PMAK2**)
	1.11	0.161	0.462	0.524	0.155				
**NB_nested MAK2**	**θ_λ_**	**θ_OVDP_**	**θ_PDV_**	**θ_PPDV_**	**ω_λ_**	**ω_PDV_**			1634.36 (−8.49 **NB_MAK2**)
	0.94	0.155	0.43	0.141	0.44	0.121			
**NB_nested nested MAK2**	**θ_λ_**	**θ_OVDP_**	**θ_PDV_**	**θ_PPDV_**	**θ_PPPDV_**	**ω_λ_**	**ω_PDV_**		1630.77 (−3.59 **NB_nested MAK2**)
	0.817	0.157	0.448	0.104	0.0955	0.401	0.0849		
**NB_nested MAK2 steroids**	**θ_λ_**	**θ_OVDP_**	**θ_PDV_**	**θ_PDV_S_**	**θ_PPDV_**	**ω_λ_**	**ω_PDV_**		1624.048 (−10.312 **NB_nested MAK2**)
	0.923	0.132	0.447	0.15	0.145	0.438	0.127		

Values between parentheses are the decreases/increases in the objective function value relative to a specified reference model.

Table 1 shows the parameter differences among models as well as the corresponding objective function values. Selected model (*NB nested MAK2*) estimates are listed in table 2 with the corresponding relative standard error (RSE %). The fixed effects parameters were estimated with adequate precision, however the RSE associated with the random effects was high as we expected (N = 9 subjects). All parameters and random effects variables for the sixteen models are listed in Table 1 with the objective function values.

**Table 2:** *NB nested MAK2* model parameters.

Parameters	Estimate	RSE(%)	ISV (CV%)	RSE(%)
**λ_0_**	0.940	24.14	66.33	60.90
**OVDP**	0.155	22.19		
**θ_PDV_**	0.430	23.72	34.78	71.57
**θ_PPDV_**	0.141	30.99		

RSE (%) relative standard error.

**Figure 3:**
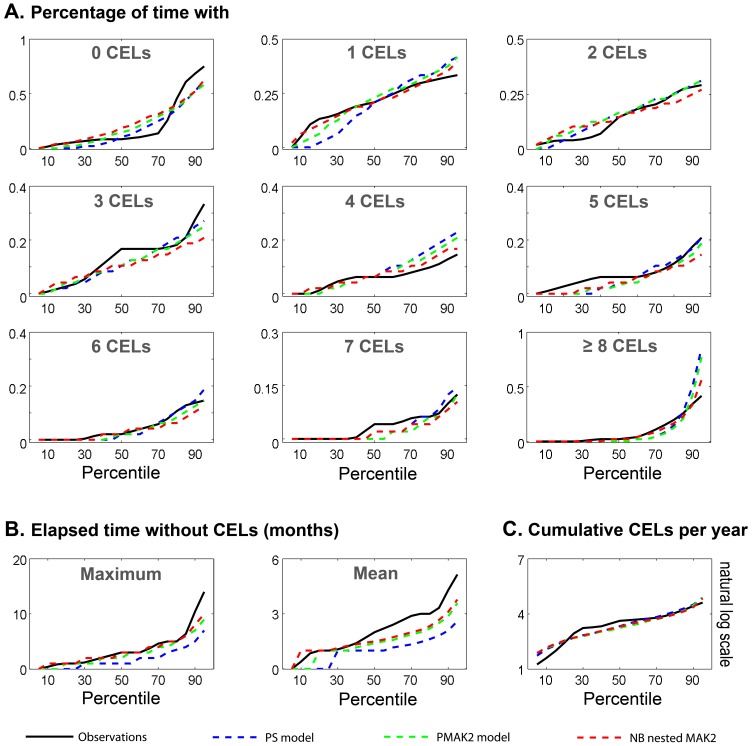
Visual Numerical Predictive Check (VNPC). Different dynamic descriptors were calculated for the observed data (black solid line) and the simulated data from the different selected models (dashed lines). Those descriptors were evaluated at different percentiles from 5^th^ to 95^th^ with an increasing step of 5.

The goodness of fit of the models was evaluated using simulation-based methods (see methods). Figure 3 shows the results for visual numerical predictive checks (VNPC) of several dynamic descriptors: *(i)* probability of having of 0, 1, 2, 3, 4, 5, 6, 7 and >8 CELs (Figure 3A), *(ii)* maximum and mean elapsed time without lesions during the four years (Figure 3 B) and *(iii)* cumulative number of CELs per year (Figure 3C). We compared the prediction performance of selected models explored in this manuscript: Poisson model (*PS*), Poisson model with first order Markov factor (*PMAK2*) and Negative Binomial model with first and second order Markov factors (*NB nested MAK2*). In general the *NB nested MAK2* performed better for most of the descriptors. None of the models adequately capture the stiff dynamic along the percentiles for the probability of having no CELs.

**Figure 4:**
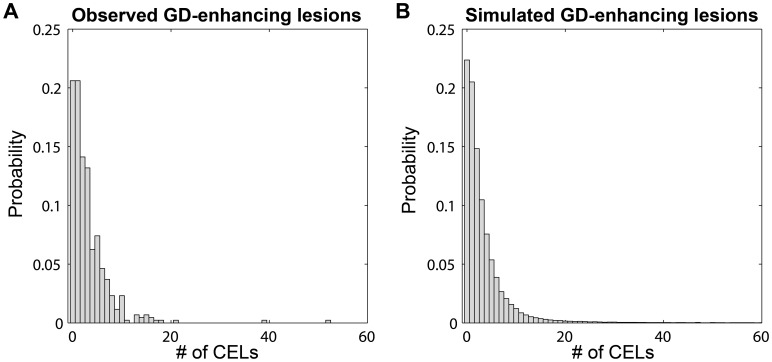
Predicted Interval of Visual Numerical Predictive Check. Different dynamic descriptors were calculated for the observed data (black solid line) and simulated data *NB nested MAK2* model. The 95% predicted interval is represented red area. Dashed red represented the simulated median. Those descriptors were evaluated at different percentiles from 10^th^ to 90^th^ with an increasing step of 5.

**Figure 5:**
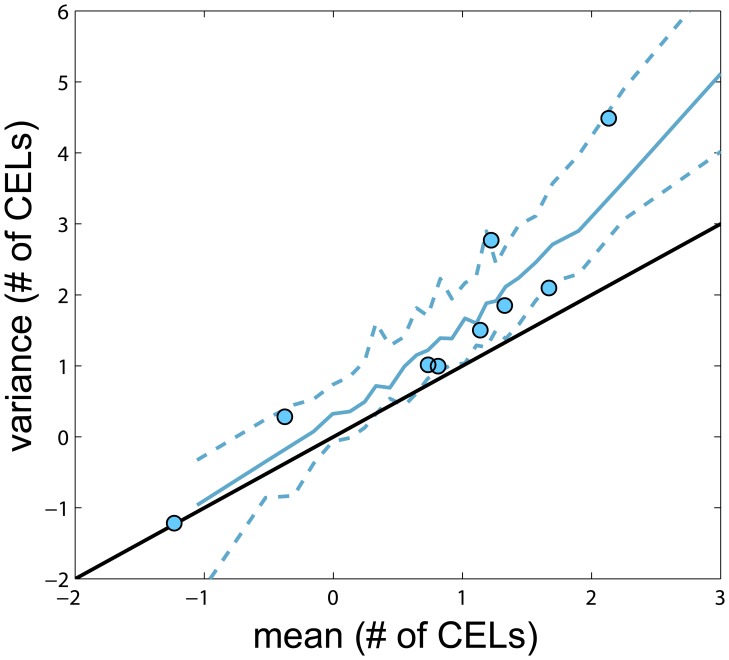
Probability distribution for CEL. Observed data **(A)** *versus* the probability distribution of simulated data **(B)** generated by *NB nested MAK2* model.

**Figure 6:**
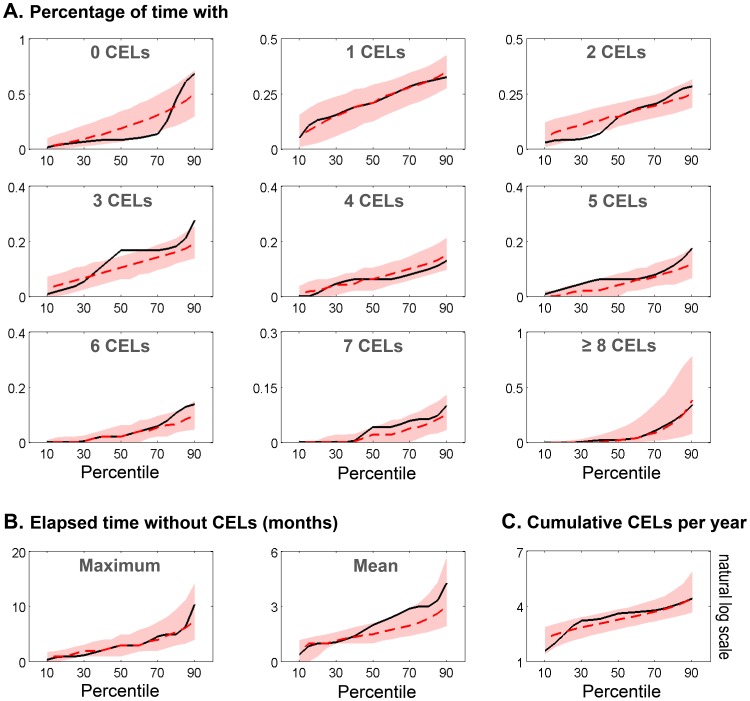
Predicted Interval for variance *versus* mean of number of CELs. Variance and mean of number of CELs in each patient (observed – simulated) were calculated and represented in natural logarithmic scale. Solid line in black corresponds to the identity line. Blue dots are the observations. Blue dashed lines correspond to the 5^th^ and 95^th^ quartiles of simulated data and solid blue line corresponds to the median of simulated data. Black solid line is the identity line.

In order to evaluate better the predictive capacity of the *NB nested MAK2*, the prediction intervals for the dynamic descriptors previously defined were calculated (Figure 4). The model captures the observed percentiles for a majority of the descriptors reasonably well. The CEL count distributions for the raw and simulated data for the selected model were also compared for model evaluation (Figure 5). Figure 6 shows the prediction interval for variance versus mean of number of CELs with the patient data. The model is able to capture the relationship between the mean number of CELs and the variance of those counts. Based on these visual model evaluations it was concluded that the *NB nested MAK2* model describes the observed data and better than other explored distributions.

**Figure 7:**
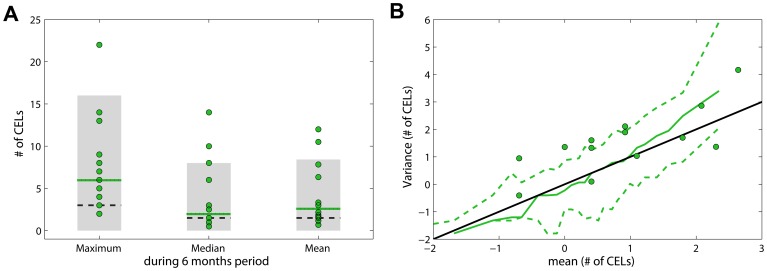
Model validation. A. Three descriptors were compared: (i) maximum, (ii) median and (iii) mean of the number of CELs during the 6 months. Green dots represented the observed data; dotted lines are the observed median; black dashed lines are the predicted median and grey areas the 95% PI by the model. B. Variance *versus* mean for a 6 time window. Green dots are observations. Green dashed lines correspond to the 5^th^ and 95^th^ percentiles of simulated data and the solid green line corresponds to the median of simulated data. Black solid line is the identity line.

The model was also validated with data from a second cohort. Model simulations for the maximum, median and mean of the number of CELs during 6 months were compared with fourteen patients with RRMS whom underwent monthly MRIs during a 6-month pre-therapy phase (Figure S1). The model captured reasonably well the median and mean although slightly under-predicted (Figure 7A). The maximum number of active lesion was clearly under-predicted (Figure 7A). The predicted interval for variance versus mean of number of CELs for a 6 month time window was slightly under-predicted for smaller means (Figure 7B). The disease activity in the group of patients used for the model validation was higher than the group used for the model building; for example, the number of CELs per patient per month in average was 4.08 and 3.26 for model validation and model building respectively.

**Figure 8:**
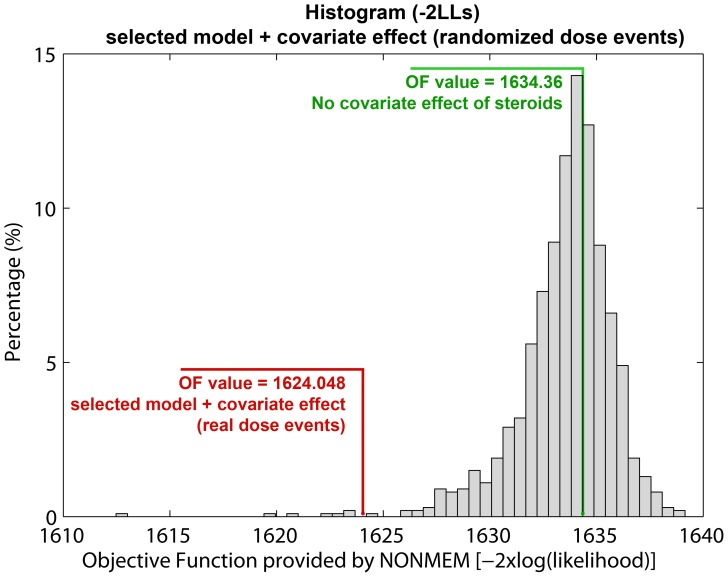
Analysis of the significance of the steroid effect by randomizing the dose events. One thousand new data files were generated by randomizing the doses event architecture while preserving the total number of dose events and the patient observations. The histogram shows the distribution of the OF values obtained using the selected model with the steroid effect when drug administrations were randomly generated. The OF value of the selected model with no steroid effect is marked in green. The OF value of the selected model with the covariate steroid effect, using the real dose moments is highlighted in red.

During the study, six patients were treated with immunomodulatory or immunosuppressive drugs (intravenous methylprednisolone at 1 g/day for 3–5 days or oral prednisone) for alleviation of clinical relapses. The months in which these patients received steroids are indicated in figure 1. A parameter for the effect of steroids was evaluated on: λ_0_, OVDP, θ_PDV_ and θ_PPDV_. Although the use of immunosuppressive drugs occurred infrequently, a steroid administration effect on the CEL events was quantifiable. A statistically significant improvement in OFV was observed when the effect was included on θ_PDV_ instead in λ_0_. This relationship was further evaluated utilizing a randomization test approach (see methods); figure 8 shows 99.3% of the randomized schemes resulted in a higher OFV, highlighting the impact of the steroid effect (p-value = 0.007). Table 3 shows the estimates with the corresponding relative standard error (RSE %) for the selected model with the steroid covariate effect. Comparing values, the parameter values for λ_0_, θ_PDV_ and θ_PPDV_ changed slightly to: −1.80%; 3.80%; and 2.75% respectively. The OVPD parameter dropped 14.86%, indicating that part of the over dispersion observed in the data might be due to the immunomodulatory treatment. The parameter θ_PDV_, is diminished 66.44% when the patient was treated with immunosuppressive drugs for that month (θ_PPDV_S_). This result suggests that the use of steroids contributes to the inflammatory resolution of persistent CELs but does not affect the new CELs generated that month. Other implications of this result are indicated in the discussion section. The steroid covariate inclusion into the model, that is a within subject effect, did not explain the ISV associated to λ_0_ and θ_PDV_. All fixed effects parameters were estimated with adequate precision except θ_PPDV_. The RSE associated with the random effects were similar to the selected model with no steroid effect. The steroid effect was also evaluated for the following month that the patient received the treatment; however no significant effect was identified.

**Table 3:** *NB nested MAK2 with steroid effects* model parameters.

Parameters	Estimate	RSE(%)	ISV (CV%)	RSE(%)
**λ_0_**	0.923	26.54	66.18	59.58
**OVDP**	0.132	25.37		
**θ_PDV_**	0.447	21.40	35.63	73.85
**θ_PDV_S_**	0.145	32.06		
**θ_PPDV_**	0.150	48.00		

RSE (%) relative standard error.

## Discussion

The disease progression of CEL dynamics is highly variable both within and between patients. In this study we identified a discrete distribution model from a pool of candidate models that best described the distribution of CELs in these patients. Although several models were able to describe the data reasonably well, the negative binomial resulted in the best fit. The identified overdispersion indicates the presence of greater intra-patient variability (variance) in the number of CELs during a period of time (48 month, 48 points) than what is expected based on the mean.

All of the models had significant improvements in fit when the information about what happened in the previous months was incorporated (i.e., Markovian elements). Nevertheless the importance of previous observations diminishes with increasing time from the observation. This may be attributable to the fact that the CEL counts noted every month were the total number of CELs, and thus, older lesions observed in previous months might persist in the current one. Working under this hypothesis, the results suggest that such persistent CELs may last up to 2 months. In other words, this indicates that although the clinical relapse (symptoms that appear in episodic acute periods) usually last less than a month, the active inflammatory event might persist for a longer time.

It is well known that the focal inflammatory events in the CNS that accompany a clinical multiple sclerosis relapse show complex dynamics. There is the potential for a great deal of insight to be generated if mechanistic elements, e.g. balance between effector and regulatory T cell, were incorporated to these kinds of probabilistic models [22]. The idea would be to identify latent variables that explain variations in the mean counts (λ). Unfortunately, with data available, we were not able to develop a more mechanistic model.

The selected model (called *NB nested MAK2*) describes the observed data well and better than other explored distributions (Figure 3, Figure 4). Although all the parameters were estimated with adequate precision, the RSE associated with the random effects were high. Therefore, the estimated values for the ISV of the parameters λ_0_ and, θ_PDV_ are not well determined. However, as shown in figure 4, the prediction intervals simulated from the model for all descriptors of CEL response captured the observed response percentiles well.

The model was externally validated with data from a second cohort. Model predictions were adequate for the median and mean numbers of events, but the maximum number of events was under-predicted. The predicted interval for variance versus mean of number of CELs was also slightly under-predicted for smaller means. These under-predictions are probably due to the disease being more active in the data set used for the validation than in the group of patients used for the model building (the average number of CELs per patient per month was 4.08 in the validation set and 3.26 in the model building set). In theory, the level of overdispersion of the 6 months simulated data should be identical to that use to simulate 48 months of data. However, when comparing both simulated data sets (6 and 48 months), we realized that this is not the case. The lower overdispersion found in 6 months simulated data is an effect of the number of months (or number of observations) within the subject. As a matter of fact, the same phenomenon also occurs if we select any 6 consecutive months of the 48 months in the observed data. Therefore, higher overdispersion is expected for both simulated and observed data when larger time windows are used for the calculation of the mean and corresponding variance of the number of active lesions.

During the course of the study, six of the nine patients were treated with corticosteroids for clinical relapses. We explored a steroid effect on all model parameters: λ_0_, OVDP, θ_PDV_ and θ_PPDV_. A significant improvement in fit was found when the effect was included on θ_PDV_ instead of λ_0_. This result suggests that the use of steroids contributes to the inflammatory resolution of persistent CELs (older CELs) but that it is not affecting to the newer CELs generated in the month after administration. Specifically, the model suggests that the use of steroids would help to resolve approximately the 66% of the persistent CELs. This result was further evaluated utilizing a randomization test strategy. One thousand randomized ensembles of the dose event architecture (see methods) were simulated to test this. Figure 8 shows the histogram of the OF values calculated (−2LL) from the simulations. 99.3% of the randomized schemes resulted in a higher OF, highlighting the statistical relevance of steroid effect (p<0.007). The steroid drug effect was evaluated not only for the month in which the patient received steroids, but also for the following month. Although a decrease for λ_0_ when patients received steroids in the previous month was identified, this result was not significant. These results reflect the utility of this modelling approach for drug effect evaluation, providing a quantitative framework that can support the informed design of future longitudinal studies and other clinical trials.

The best probabilistic model, fitting the distribution of CELs developed by nine MS patients undergoing monthly MRI evaluations over 48 months, was developed. The information that can be extracted from this kind of count data depends on the resolution with which data are collected as well as the coincidence of the measurement paradigm with the CEL cycle. Other approach/methodologies have been applied with relative similar purposes [17]–[21], [23]–[24]. Although in general the number of patients analysed in those studies was larger; the recording timings for the MRIs was not of sufficient resolution for capturing the CELs dynamic. The short interval between MRI acquisitions (one month) provides an appealing time resolution to capture the relapsing-remitting complex dynamics of the CELs. In this data analysis, an additional step was taken by applying the nonlinear mixed effect modelling approach. This provides a quantitative analysis of the data allowing the incorporation and quantification of both fixed and random effects. This approach takes into account the information from all patients simultaneously, defining both the population tendency for each parameter and the inter-patient variability in that parameter. This methodology has been widely applied and evaluated in research fields such as pharmacometrics. It is an approach that is especially well suited for the analysis of repeated measurements. The selected model was comprehensively evaluated (OFV comparisons, goodness of fit plots, visual/numerical predictive checks, parameter precision…) and externally validated using data from a second cohort.

## Materials and Methods

### Patients and MRI Scans

The study was performed at the National Institutes of Health in Bethesda, MD, USA. The Intramural Research Board of the National Institute of Neurological Disorders and Stroke approved the study. Informed written consent was obtained from each patient. The MRIs were performed on a 1.5-T magnet (General Electric Medical Systems, Milwaukee, Wisconsin) using a standard head coil as previously described. At each monthly MRI, the total number of contrast-enhancing lesions (CELs) on T1-weighted post-contrast scans was identified by experienced radiologists (Figure 1). Clinical and imaging details about this cohort are described in detail elsewhere [25]. Nine patients with MS were sequentially enrolled. Patients were enrolled in the study if they had never been treated with immunomodulatory or immunosuppressive drugs, except for intravenous methylprednisolone at 1 g/day for 3–5 days, or oral prednisone taper for a clinical relapse. In addition, patients were required to be able to complete monthly MRI scans and to have been steroid-free for at least 1 month at study entry. After a complete neurological examination, including rating disability using the Expanded Disability Status Score (EDSS) and initial MRI scan at baseline, patients were subsequently examined and imaged monthly. The number of total CELs in each month was calculated as the sum of all the CELs that were enhancing at that month for the last time. Thus, each CEL considered for the analysis was counted only once.

### Data Analysis

Data were analysed employing the population approach using the Laplacian integral approximation method implemented in the software NONMEM version VII (Icon Development Solutions, Hanover, Maryland).

### Models for Count Data

Sixteen models based on seven different probability distributions were explored: *(i)* Poisson model, *(ii)* Poisson model with Markov elements, *(iii)* Poisson model with mixture distribution, *(iv)* Zero-Inflated Poisson model, *(v)* Generalized Poisson model, *(vi)* Negative Binomial model and *(vii)* Zero-Inflated Negative Binomial model.

- Poisson model (PS). The Poisson distribution is used to model the number of events occurring within a given time interval. The PS model is expressed by the probability *P* (likelihood) that the random variable *Y* is equal to the number of counts *n* (equation 1). The parameter lambda λ represents the mean number of counts in a given time period and is the unique parameter of this model. The use of a PS model implies the assumption of equi-dispersion, meaning equality between the mean and the variance of the data (equation 2). Another important assumption of this model is that the number of counts (λ) occurring in non-overlapping intervals of time is independent.

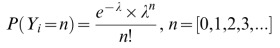
(1)

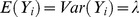
(2)
- Poisson model with Markov elements (PMAK). The Markovian element provides for the dependence of events across time-points. Markov elements were explored for Poisson model in two different ways: PMAK1 and PMAK2. Stating that an observation is conditional on the previous one, and not the one before, is permitted by the inclusion of a first order Markovian component. Higher orders were also incorporated.
  - PMAK1 model (equation 3). A binary covariate PDV_1_ was included in the data file taking the value 1 or 0 depending on whether there was at least one CEL or not at the previous month 
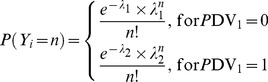
(3)
  - PMAK2. The covariate PDV was created and incorporated taking the value of the previous dependent variable. In this case, the parameter λ is modified by the PDV term (equation 4). Higher orders were also explored: second (equation 5) and third (equation 6) order Markovian component, called *nested PMAK2* and *nested nested PMAK2* respectively.

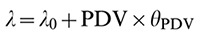
(4)

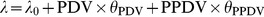
(5)

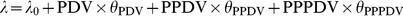
(6)
- The Poisson model utilizing a mixture distribution for individual observations (PMIX) (equation 7) incorporates an additional parameter, the mixture probability (MP) for an observation to belong to one of the two mixture distribution (characterized by two different λs) within an individual [26]:
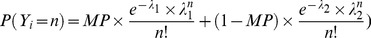
(7)
- Zero-Inflated Poisson model (ZIP) is a mixture model adapted for data presenting an excess of zero values and therefore needing an adaptation of the distribution that is otherwise extremely skewed. ZIP is a special case of PMIX where λ_1_ is equal to 0. It is composed of two equations depending on whether the random variable is a zero or a greater value and they include the probability P_0_ of the observation being zero (equation 8). If P_0_ is equal to zero, the ZIP model reduces to the PS model. The mean monthly CEL count and the variance will be given by the following expressions [(1 – P_0_) ×λ] and [(1 – P_0_) ×λ× (1+ P_0_×λ)], respectively.
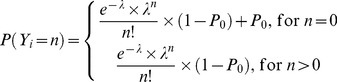
(8)
- Generalized Poisson model (GP) (equation 9) possesses the twin property of over dispersion and under dispersion [27]. This is contained in the dispersion parameter δ that can be positive or negative within the adaptive range [max (−1, −λ/4), 1]. First (equation 5) and second (equation 6) order Markov elements were explored for this distribution models affecting to the λ parameter, called GP PMAK2 and GP nested PMAK2 respectively.
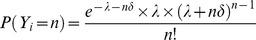
(9)
- Negative Binomial model (NB) is used when there is over dispersion due to latent heterogeneity (equation 10). The NB model is a mixture of the Poisson distribution when the mean follows a Gamma distribution [28] and is a function of λ and a parameter which accounts for the degree of over dispersion called here OVDP. The mean is still λ, but the variance becomes λ×(1-OVDP×λ). As OVDP approaches zero the NB model approaches the PS model. OVPD is restricted to be positive. First (equation 4), second (equation 5) and third (equation 6) order Markov elements were explored for this distribution models affecting to the λ parameter, called NB PMAK2, NB nested PMAK2 and NB nested nested PMAK2 respectively.
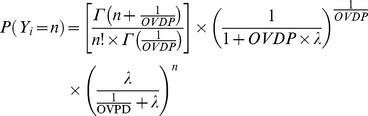
(10)
- Zero-Inflated Negative Binomial model (ZINB). Equation 11 corresponds to the zero-inflated negative binomial. The parameters P_0_ and OVDP have the same meaning as in the ZIP model. The ZINB model reduces to the ZIP, NB or PS model when P_0_, OVDP or both approaches zero, respectively
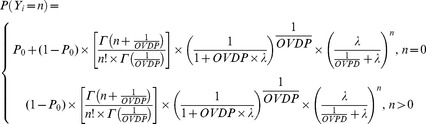
(11)Once the basic structure of the model was identified, the effect of the steroid treatments on the model parameters was evaluated. This was done for every model parameter, i.e., λ_0_, OVDP, θ_PDV_ and θ_PPDV_. The drug effect was evaluated not only for the month in which the patient received steroids, but also for the following month to evaluate longer-term or delayed effects.

### Model Development and Selection Criteria

The minimum value of the objective function (OFV) provided by NONMEM, which corresponds approximately to −2×log(likelihood) [−2LL], served as a criteria for model comparison during the model development process. A decrease in −2LL of 6.63 points for one additional parameter, was regarded as a significant model improvement corresponding to p-value of 0.01 for nested models. The Akaike information criteria (AIC), calculated as AIC = −2LL+2×NP, where NP is the number of parameter in the model, was used for selection among non-nested models [29]. The choice of the final model was based also on the OFV value, the precision of parameter estimates, and the results from model predictive performance where raw data and data obtained from model simulations were compared.

### Model Evaluation

Models were evaluated in more detail as follows: *(i)* Visual Numerical Predictive Checks (VNPC). One thousand individuals were simulated using the selected models and their model parameter estimates. For the observed data and each simulated dataset the following descriptors were calculated: probability of having of 0, 1, 2, 3, 4, 5, 6, 7 and >8 CELs, maximum and mean elapsed time without lesions during the four years and number of cumulative CELs per year. For every descriptor, the increasing percentiles from 5^th^ to 95^th^ were calculated. The results for the raw and simulated data with the different models were plotted where X axis represent the different percentiles (Figure 3). *(ii)* Predicted interval of VNPC. One thousand studies with 9 individuals per study were simulated using the selected model. The same dynamic descriptors that were described for the VNPC were used here. For every descriptor, the increasing percentiles from 10^th^ to 90^th^ were calculated. The 95% prediction interval by model was plotted with the data (Figure 4). *(iii)* Probability CEL distribution; observed data was compared to the probability distribution of simulated data generated by the selected model (Figure 5). *(iv)* Predicted interval for variance versus mean of number of CELs; one thousand simulated individuals with the selected model were generated. The individual mean CELs counts and the individual variance for every patient were computed from the raw data. The same computations were then made for each simulated individual and year; for a total of 1000. The results were divided into 20 intervals for the mean of CELs, with each interval containing 50 simulated subjects. For each interval, variances were binned, and the median and 5^th^–95^th^ percentiles were calculated. Finally, the overall median and percentiles were represented graphically together with those corresponding to the raw data (Figure 6).

### Model Validation

Fourteen patients with relapsing-remitting multiple sclerosis underwent monthly MRIs during a 6-month pre-therapy phase. None of these patients were treated with any immunosuppressive therapy before the first scan. On each MRI, the total number of CELs was noted (Figure S1). This open-label study was performed at the National Institutes of Health, Bethesda, Maryland, with approval from the institutional review board [30]. Three descriptors were used for model validation (Figure 7A): (i) maximum number of CELs, (ii) median of the CEL counts and (iii) mean of the number of CELs collected during the 6 months pre-therapy phase. Moreover, the predicted interval for variance versus mean number of CELs for a 6 time window by the selected model was compared with the same values from the data set (Figure 7B).

### Randomization of the Steroid Dose Administrations

A randomization test to calibrate the false positive rate for the evaluation of the steroid effect was conducted. Specifically, one thousand new data files were generated by randomizing the dose event architecture while preserving the total number of dose events and the patient observations.

## Supporting Information

Figure S1 **Cohort for model validation.** CEL counts are represented with circles and dashed lines (left Y axis).
(TIF)

## References

[1] Compston A, Coles A (2008) Multiple sclerosis. Lancet 372: 1502–1517. 18970977 10.1016/S0140-6736(08)61620-7

[2] Vollmer T (2007) The natural history of relapses in multiple sclerosis. J Neurol Sci 256 Suppl 1S5–13. 17346747 10.1016/j.jns.2007.01.065

[3] Paty DW, McFarland H (1998) Magnetic resonance techniques to monitor the long term evolution of multiple sclerosis pathology and to monitor definitive clinical trials. J Neurol Neurosurg Psychiatry 64 Suppl 1S47–51. 9647285

[4] Miller DH (2002) MRI monitoring of MS in clinical trials. Clin Neurol Neurosurg 104: 236–243. 12127661 10.1016/s0303-8467(02)00045-8

[5] Bakshi R, Thompson AJ, Rocca MA, Pelletier D, Dousset V, et al (2008) MRI in multiple sclerosis: current status and future prospects. Lancet Neurol 7: 615–625. 18565455 10.1016/S1474-4422(08)70137-6 PMC2586926

[6] Bastianello S, Pozzilli C, Bernardi S, Bozzao L, Fantozzi LM, et al (1990) Serial study of gadolinium-DTPA MRI enhancement in multiple sclerosis. Neurology 40: 591–595. 2320230 10.1212/wnl.40.4.591

[7] McFarland HF, Stone LA, Calabresi PA, Maloni H, Bash CN, et al (1996) MRI studies of multiple sclerosis: implications for the natural history of the disease and for monitoring effectiveness of experimental therapies. Mult Scler 2: 198–205. 9345374 10.1177/135245859600200406

[8] Confavreux C, Vukusic S, Moreau T, Adeleine P (2000) Relapses and progression of disability in multiple sclerosis. N Engl J Med 343: 1430–1438. 11078767 10.1056/NEJM200011163432001

[9] Martin R, McFarland HF, McFarlin DE (1992) Immunological aspects of demyelinating diseases. Annu Rev Immunol 10: 153–187. 1375472 10.1146/annurev.iy.10.040192.001101

[10] Hauser SL, Oksenberg JR (2006) The neurobiology of multiple sclerosis: genes, inflammation, and neurodegeneration. Neuron 52: 61–76. 17015227 10.1016/j.neuron.2006.09.011

[11] Miller R, Frame B, Corrigan B, Burger P, Bockbrader H, et al (2003) Exposure-response analysis of pregabalin add-on treatment of patients with refractory partial seizures. Clin Pharmacol Ther 73: 491–505. 12811359 10.1016/S0009-9236(03)00049-3

[12] Jonker DM, Voskuyl RA, Danhof M (2004) Pharmacodynamic analysis of the anticonvulsant effects of tiagabine and lamotrigine in combination in the rat. Epilepsia 45: 424–435. 15101823 10.1111/j.0013-9580.2004.50503.x

[13] Gupta SK, Sathyan G, Lindemulder EA, Ho PL, Sheiner LB, et al (1999) Quantitative characterization of therapeutic index: application of mixed-effects modeling to evaluate oxybutynin dose-efficacy and dose-side effect relationships. Clin Pharmacol Ther 65: 672–684. 10391673 10.1016/S0009-9236(99)90089-9

[14] Godfrey CJ (2007) Mixed effects modelling analysis of count data. In: Ette EI, Willliams PJ (eds) Pharmacometrics: the science of quantitative pharmacology. Wiley-Interscience, New York.

[15] Troconiz IF, Plan EL, Miller R, Karlsson MO (2009) Modelling overdispersion and Markovian features in count data. J Pharmacokinet Pharmacodyn 36: 461–477. 19798550 10.1007/s10928-009-9131-y

[16] Plan EL, Maloney A, Troconiz IF, Karlsson MO (2009) Performance in population models for count data, part I: maximum likelihood approximations. J Pharmacokinet Pharmacodyn 36: 353–366. 19653080 10.1007/s10928-009-9126-8

[17] Sormani MP, Bruzzi P, Miller DH, Gasperini C, Barkhof F, et al (1999) Modelling MRI enhancing lesion counts in multiple sclerosis using a negative binomial model: implications for clinical trials. J Neurol Sci 163: 74–80. 10223415 10.1016/s0022-510x(99)00015-5

[18] Sorman MP, Bruzzi P, Rovaris M, Barkhof F, Comi G, et al (2001) Modelling new enhancing MRI lesion counts in multiple sclerosis. Mult Scler 7: 298–304. 11724445 10.1177/135245850100700505

[19] Sormani MP, Miller DH, Comi G, Barkhof F, Rovaris M, et al (2001) Clinical trials of multiple sclerosis monitored with enhanced MRI: new sample size calculations based on large data sets. J Neurol Neurosurg Psychiatry 70: 494–499. 11254773 10.1136/jnnp.70.4.494 PMC1737302

[20] van den Elskamp I, Knol D, Uitdehaag B, Barkhof F (2009) The distribution of new enhancing lesion counts in multiple sclerosis: further explorations. Mult Scler 15: 42–49. 18845655 10.1177/1352458508096683

[21] Healy BC, Ikle D, Macklin EA, Cutter G (2010) Optimal design and analysis of phase I/II clinical trials in multiple sclerosis with gadolinium-enhanced lesions as the endpoint. Mult Scler 16: 840–847. 20530124 10.1177/1352458510371409

[22] Velez de Mendizabal N, Carneiro J, Sole RV, Goni J, Bragard J, et al (2011) Modeling the effector - regulatory T cell cross-regulation reveals the intrinsic character of relapses in Multiple Sclerosis. BMC Syst Biol 5: 114. 21762505 10.1186/1752-0509-5-114 PMC3155504

[23] Meier DS, Guttmann CR (2006) MRI time series modeling of MS lesion development. Neuroimage 32: 531–537. 16806979 10.1016/j.neuroimage.2006.04.181

[24] Sormani M, Stromillo ML, Battaglini M, Signori A, De Stefano N (2012) Modelling the distribution of cortical lesions in multiple sclerosis. Mult Scler 18: 229–231. 21757533 10.1177/1352458511414965

[25] Bagnato F, Jeffries N, Richert ND, Stone RD, Ohayon JM, et al (2003) Evolution of T1 black holes in patients with multiple sclerosis imaged monthly for 4 years. Brain 126: 1782–1789. 12821527 10.1093/brain/awg182

[26] Wang P, Puterman ML, Cockburn I, Le N (1996) Mixed Poisson regression models with covariate dependent rates. Biometrics 52: 381–400. 10766499

[27] Yang Z, Hardin JW, Addy CL, Vuong QH (2007) Testing approaches for overdispersion in poisson regression versus the generalized poisson model. Biom J 49: 565–584. 17638291 10.1002/bimj.200610340

[28] Winkelmann R, Zimmermann KF (1994) Count data models for demographic data. Math Popul Stud 4: 205–221, 223. 10.1080/08898489409525374 12287090

[29] Ludden TM, Beal SL, Sheiner LB (1994) Comparison of the Akaike Information Criterion, the Schwarz criterion and the F test as guides to model selection. J Pharmacokinet Biopharm 22: 431–445. 7791040 10.1007/BF02353864

[30] Chiu AW, Richert N, Ehrmantraut M, Ohayon J, Gupta S, et al (2009) Heterogeneity in response to interferon beta in patients with multiple sclerosis: a 3-year monthly imaging study. Arch Neurol 66: 39–43. 19001157 10.1001/archneur.66.1.noc80047

