# Correction: Predicting Relapsing-Remitting Dynamics in Multiple Sclerosis Using Discrete Distribution Models: A Population Approach

**DOI:** 10.1371/annotation/40be1d29-5c85-40a8-a379-666ffd18afdc

**Published:** 2014-01-06

**Authors:** Nieves Velez de Mendizabal, Matthew M. Hutmacher, Iñaki F. Troconiz, Joaquín Goñi, Pablo Villoslada, Francesca Bagnato, Robert R. Bies

Figures 4, 5, and 6 were incorrectly switched. The image currently appearing as Figure 4 belongs with the title and legend for Figure 5, the image currently appearing as Figure 5 belongs with the title and legend for Figure 6, and the image currently appearing as Figure 6 belongs with the title and legend for Figure 4. The titles and legends for the given figures are in correct order. 

