# Supplementary figures and images for: Predicting Relapsing-Remitting Dynamics in Multiple Sclerosis Using Discrete Distribution Models: A Population Approach
Source: PLoS One. 2013 Sep 5;8(9):e73361. doi: 10.1371/journal.pone.0073361 (PMC3764125; doi:10.1371/journal.pone.0073361)

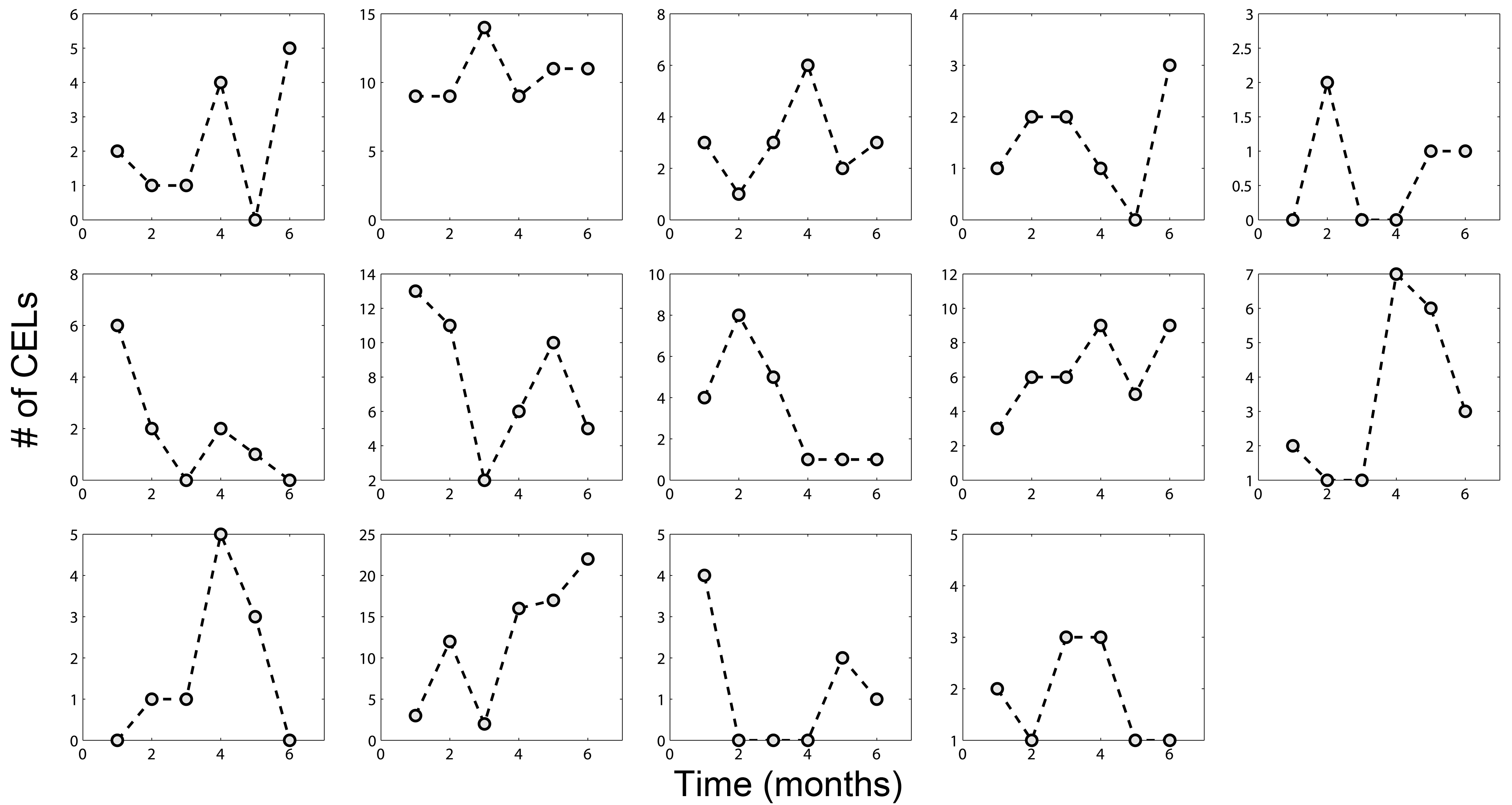

Supplement: Figure S1 — Cohort for model validation. CEL counts are represented with circles and dashed lines (left Y axis). (TIF) [file pone.0073361.s001.tif]
